# Diabetes Mellitus and Risk of Bladder Cancer: A Meta-Analysis of Cohort Studies

**DOI:** 10.1371/journal.pone.0058079

**Published:** 2013-03-05

**Authors:** Xin Xu, Jian Wu, Yeqing Mao, Yi Zhu, Zhenghui Hu, Xianglai Xu, Yiwei Lin, Hong Chen, Xiangyi Zheng, Jie Qin, Liping Xie

**Affiliations:** Department of Urology, First Affiliated Hospital, Zhejiang University, Hangzhou, Zhejiang Province, China; CUNY, United States of America

## Abstract

**Objective:**

Diabetes is associated with increased risk of cancer at several sites, but its association with risk of bladder cancer is still controversial. We examined this association by conducting a systematic review and meta-analysis of cohort studies.

**Methods:**

Studies were identified by searching PubMed, EMBASE, Scopus, Web of Science, Cochrane register, and Chinese National Knowledge Infrastructure (CNKI) databases through April 29, 2012. Summary relative risks (SRRs) with their corresponding 95% confidence intervals (CIs) were calculated using a random-effects model.

**Results:**

A total of fifteen cohort studies were included in this meta-analysis. Analysis of all studies showed that diabetes was associated with a borderline statistically significant increased risk of bladder cancer (RR 1.11, 95% CI 1.00–1.23; p<0.001 for heterogeneity; I^2^ = 84%). When restricting the analysis to studies that had adjusted for cigarette smoking (n = 6) or more than three confounders (n = 7), the RRs were 1.32 (95% CI 1.18–1.49) and 1.20 (95% CI 1.02–1.42), respectively. There was no significant publication bias (p = 0.62 for Egger’s regression asymmetry test).

**Conclusions:**

Our findings support that diabetes was associated with an increased risk of bladder cancer. More future studies are warranted to get a better understanding of the association and to provide convincing evidence for clinical practice in bladder cancer prevention.

## Introduction

Urinary bladder cancer ranks ninth in worldwide cancer incidence. It is the seventh most common malignancy in men and seventeenth in women [1]. An estimated 386,300 new cases and 150,200 deaths from bladder cancer occurred in 2008 worldwide. The highest incidence rates are found in the countries of Europe, North America, and Northern Africa [2]. Increasing evidence suggests a significant influence of genetic predisposition on bladder incidence [3]; the role of genetic factors in the etiology of bladder cancer is estimated to be about 31% [4]. Cigarette smoking, occupational exposure to arylamines, and schistosomal infection are the most established external risk factors for bladder cancer [5]. However, other independent risk factors are not clearly known and their roles in bladder cancer severity, progression and outcomes need further exploration.

Over the past few decades, the prevalence of diabetes mellitus has increased substantially and is highly suspected to be associated with an increased risk of some cancers. Considerable epidemiological studies and systematic reviews have shown positive associations between diabetes mellitus and the risk of biliary tract cancer [6], liver cancer [7], kidney cancer [8], pancreas cancer [9], and colon and rectal cancer [10]. Likewise, relationships between diabetes and bladder cancer incidence have also been evaluated, yielding controversial results. Most studies have reported positive, but nonsignificant associations, which might be explained by the insufficient statistical power of individual studies.

A meta-analysis of the association between diabetes and bladder cancer risk published in 2006 concluded that diabetes was significantly associated with a higher risk (24%) of bladder cancer [11]. However, some limitations of this meta-analysis have to be mentioned, including a mixture of case–control and cohort studies, a mixture of bladder cancer incidence and mortality, lack of differentiation between type 1 and type 2 diabetes, and small numbers of bladder cancer case in most included studies. Since then, there are also many high-quality cohort studies on this association have been published [12]–[21], but controversy still reigns.

Given the inconsistency of the existing literature and the insufficient statistical power of primary studies, we performed a meta-analysis of all eligible cohort studies to derive a more precise estimation of the relationship between diabetes and risk of bladder cancer. Furthermore, we also examined whether the association between them differs according to various study characteristics.

## Materials and Methods

### Publication Search

We carried out a search in PubMed, EMBASE, Scopus, Web of Science, Cochrane register, and Chinese National Knowledge Infrastructure (CNKI) databases, covering all the papers published from their inception to April 2012. The search strategy included terms for outcome (bladder neoplasm or bladder cancer or bladder tumor) and exposure (diabetes or diabetes mellitus). We evaluated potentially relevant publications by examining their titles and abstracts and all the studies matching the eligible criteria were retrieved. We also checked the references from retrieved articles and reviews to identify any additional relevant study.

### Inclusion Criteria

Studies included in this meta-analysis had to meet all the following criteria: (a) they had a cohort design or nested case-control design; (b) one of the exposure of interest was diabetes mellitus; (c) one of the outcome of interest was incidence of bladder cancer; and (d) studies provided rate ratio, hazard ratio or standardized incidence ratio (SIR) with their 95% CIs, or data to calculate them. Studies on mortality rates from bladder cancer were not included, as it could be confounded by survival related factors. We also did not consider studies in which the exposure of interest was mainly or solely type 1 diabetes, which was defined as early-onset (age <30 years) of diabetes. If multiple publications from the same study population were available, the most recent and detailed study was eligible for inclusion in the meta-analysis.

### Data Extraction

Data were extracted independently by two authors using a predefined data collection form, with disagreements being resolved by consensus. For each study, the following characteristics were collected: first author’s name, year of publication, the country in which the study was carried out, participant characteristics (age and gender), year of study conducted, range for follow-up, sample size (cases and cohort size), methods of ascertainment of diabetes and bladder cancer, estimate effects with their 95% CIs, and covariates adjusted for in the analysis. From each study, we extracted the RR estimate that was adjusted for the greatest number of potential confounders.

### Statistical Methods

Studies that reported different measures of RR were included in this meta-analysis: rate ratio, hazard ratio and SIR. In practice, these three measures of effect yield similar estimates of RR because the absolute risk of bladder cancer is low.

Summary RR estimates with their corresponding 95% CIs were calculated with the DerSimonian and Laird [22] random effects models, which consider both within-study and between-study variation. Subgroup analyses were carried out by (a) geographic region, (b) smoking status, (c) the number of covariates adjusted for, (d) methods of ascertainment of diabetes. Only the studies based on rate ratio or hazard ratio were included for subgroup analysis.

Homogeneity of RRs across studies was tested by Q statistic (significance level at P<0.10) and the I^2^ score. Publication bias was assessed using Begg’s test (rank correlation method) [23] and Egger’s test (linear regression method) [24]. P<0.05 was considered to be representative of a significant statistical publication bias. All of the statistical analyses were performed with STATA 11.0 (StataCorp, College Station, TX), using two-sided P-values.

## Results

### Literature Search

**Figure 1:**
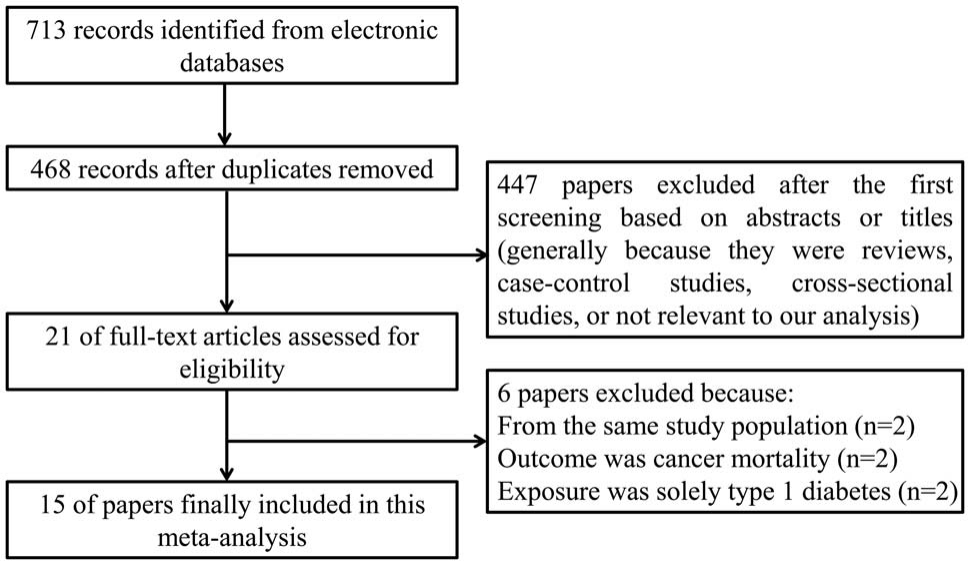
Flowchart of study assessment and selection.

Figure 1 outlines our study selection process. Briefly, after removing duplications, the search strategy generated 468 articles. Of these, the majority were excluded after the first screening based on abstracts or titles, mainly because they were reviews, case-control studies, cross-sectional studies, or not relevant to our analysis.

After full-text review of 21 papers, 6 studies were excluded for the reasons as follows: overlapping publications from the same study population [25], [26]; the outcome was cancer mortality [27], [28]; the exposure was solely type 1 diabetes [29], [30]. Thus, a total of 15 cohort studies, which met the inclusion criteria, were included in this meta-analysis.

### Study Characteristics

The characteristics of the 15 cohort studies are presented in Tables 1 and 2. Of these, 10 studies used rate ratio or hazard ratio as the measurement of RR [12]–[15], [17], [18], [20], [21], [31], [32] (Table 1), and 5 cohort studies used standardized incidence ratio as the measurement of RR [16], [19], [33]–[35] (Table 2). The studies were conducted in the following regions: Europe (n = 7) [14]–[16], [19], [21], [33], [35], Asia (n = 4) [12], [13], [20], [31], and USA (n = 4) [17], [18], [32], [34]. The study population in ten studies consisted of both sexes [12], [15], [16], [18]–[21], [33]–[35], four studies included men only [13], [14], [17], [31] and one study included women only [32]. All included studies were published between 1982 and 2012, of which 66.7% (n = 10) [12]–[21] were published in 2006 or more recent years, and were not included in previous meta-analysis. The cohort ranged in size from 1,135 [34] to 4,501,578 [17]. Diabetes status was ascertained by self-reported history of diabetes mellitus, medical records or blood glucose level. Diagnosis of bladder cancer was based on medical record or cancer registry data, except one using histological verification [34]. Adjustments were made for potential confounders of one or more factors in all studies.

**Table 1:** Characteristics of cohort studies of diabetes and bladder cancer based on rate ratio and hazard ratio.

Study	Year of study conducted	Follow up, years	Age/gender	Cases/Cohort	Diabetes assessment	Bladder cancer ascertainment	Adjustments
Lo et al./2012	1996–2009	3.5	All ages	4,311/1,790,868	Medical records	Cancer registry	Sex, age, urbanization, hypertension and hyperlipidemia.
(Taiwan)			Male: 49.1%		(type 2)		
Attner et al./2012	1998–2007	10	45–84 (86%)	19,756/167,080	Medical records	Cancer registry	Age and gender
(Sweden)			Male: 53%		(type 1 and 2)		
Atchison et al./2011	1969–1996	10.5	18–100	19,300/4,501,578	Medical records	Medical records	Age, time, latency, race, number of hospital visits, alcohol-related
(USA)			Male: 100%		(type 2)		conditions, obesity and chronic obstructive pulmonary disease
Woolcott et al./2011	1993–2004	10.7	45–75	818/185,816	Self-reported	Cancer registry	Ethnicity, sex, smoking status, intensity and duration, and employment in a
(USA)			Male: 45%		(type 1 and 2)		high risk Industry
Ogunleye et al./2009	1993–2004	3.9	All ages	68/9,577	Medical records	Cancer registry	Age, sex and deprivation
(Scotland, UK)			Male: 53%		(type 2)		
Larsson et al./2008	1997–2007	9.3	45–79	414/45,906	Self-reported	Cancer registry	Age, education, smoking status and pack-years of smoking
(Sweden)			Male: 100%		(type 1 and 2)		
Inoue et al./2006	1988–1999	14	40–69	135/97,771	Self-reported	Cancer registry	Age, study area, history of cerebrovascular disease, history of ischemic
(Japan)			Male: 47.6%		(type 1 and 2)		heart disease, smoking, physical activity, BMI, alcohol intake, green
							vegetable intake, coffee
Khan et al./2006	1988–1997	18–20	40–79	60/56,881	Self-reported	Cancer registry	Age, smoking, BMI and alcohol
(Japan)			Male: 41%		(type 1 and 2)		
Jee et al./2005	1992–2002	10	30–95	NA/829,770	Blood glucose level or	Cancer registry and	Age, smoking and alcohol
(Korea)			Male: 64%		medication use (type 2)	medical records	
Tripathi et al./2002	1986–1998	13	55–69	112/37,459	Self-reported	Cancer registry	Age, smoking, regular physical activity, BMI, alcohol, married, occupation
(USA)			Male: 0%		(type 1 and 2)		lifetime

NA, data not applicable; BMI, body mass index.

**Table 2:** Characteristics of cohort studies of diabetes and bladder cancer based on standardized incidence ratio.

Study	Year of studyconducted	Follow up,years	Age/gender	Cases/Cohort	Diabetes assessment	Bladder cancerascertainment	Adjustments
Wotton et al./2011	1963–2008	NA	≥30	2,385/484,356	Medical records	Medical records	Sex, age in 5-year bands, time period in single calendar years
(UK)			Male:54%		(estimated 90% type 2)		and district of residence
Hemminki et al./2010	1964–2007	15	>39	483/125,126	Medical records	Cancer registry	Age, sex, period, region and socioeconomic status
(Sweden)			Male: NA		(type 2)		
Swerdlow et al./2005	1972–2003	18	30–49	20/5,066	Self-reported	Cancer registry	Age, sex, country of residence and calendar year
(UK)			Male:58.1%		(estimated 36% type 1, 64% type 2)		
Wideroff et al./1997	1977–1989	17	64(m); 69(f)	493/109,581	Medical records	Cancer registry	Age, sex and calendar year
(Denmark)			Male: 49%		(type 1 and 2)		
Ragozzino et al./1982	1945–1969	8.6	61	7/1,135	Blood glucose level	Histological verification	Age
(USA)			Male: 53%		(NA)		

NA, data not applicable; m, male; f, female.

### Diabetes Mellitus and Risk of Bladder Cancer

**Figure 2:**
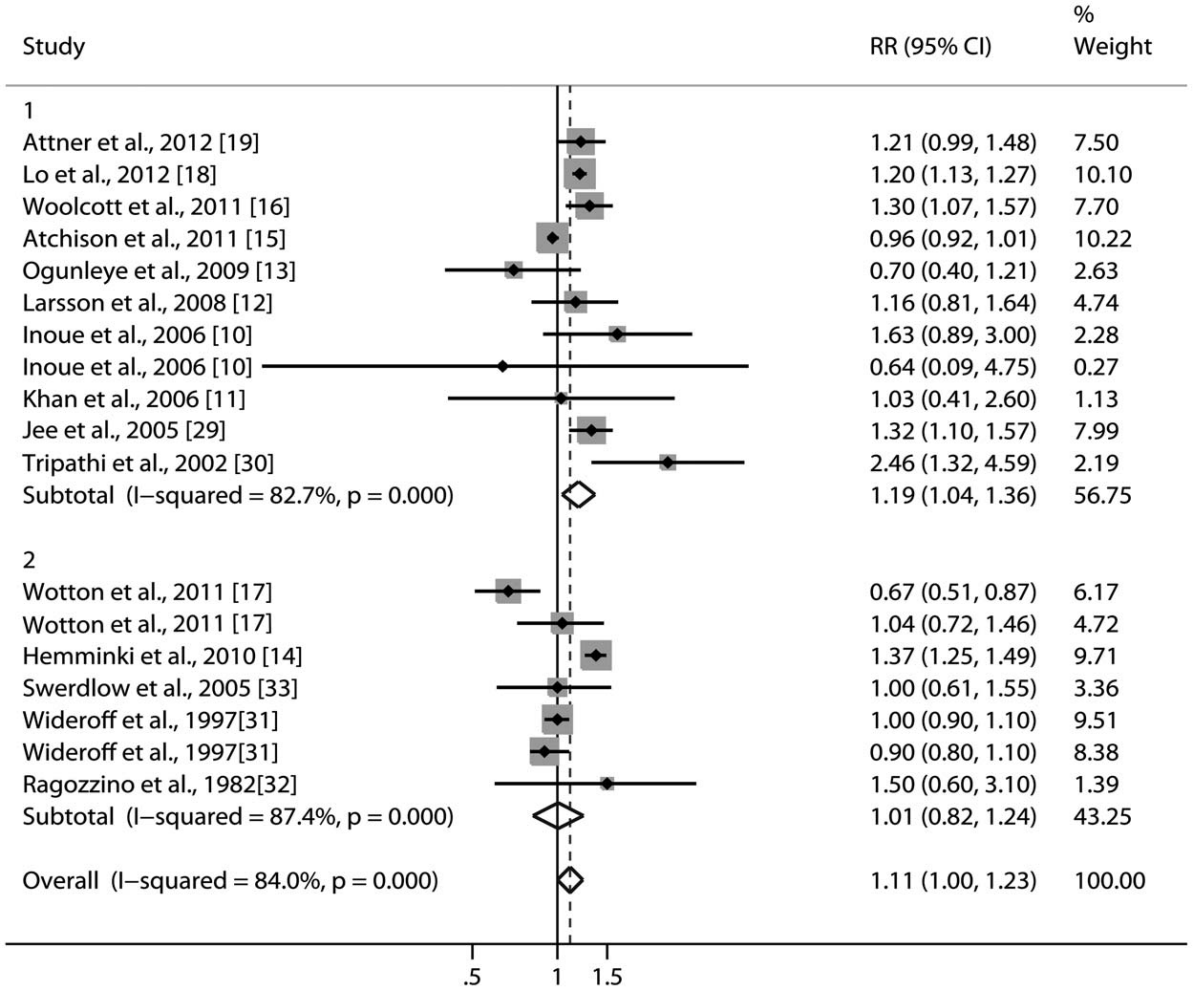
Relative risks for the association between diabetes and risk of bladder cancer in cohort studies. Studies are sub-grouped according to the measurements of relative risk. Diamonds represent study-specific relative risks or summary relative risks with 95% CIs; horizontal lines represent 95% confidence intervals (CIs). Test for heterogeneity among studies: p<0.001, I^2^ = 84.0%. 1, cohort studies (n = 10) use incidence rate as the measurement of relative risk. 2, cohort studies (n = 5) use standardized incidence rate as the measurement of relative risk.

The overall RR with its 95% CI showed a borderline statistically significant association between diabetes mellitus and risk of bladder cancer (Fig. 2, RR 1.11, 95% CI 1.00–1.23). The summary RRs with 95% CIs were 1.01 (95% CI 0.82–1.24) for studies using standardized incidence ratio, and 1.19 (95% CI 1.04–1.36) for rate ratio or hazard ratio. There was statistically significant heterogeneity among studies (p<0.001 for heterogeneity; I^2^ = 84.0%).

Next, we conducted subgroup meta-analysis by various study characteristics (Table 3). In the subgroup analysis by geographical area, the association between diabetes and bladder cancer was more significant for studies conducted in Asia (RR 1.21, 95% CI 1.15–1.28; p = 0.658 for heterogeneity; I^2^ = 0%) than in Europe (RR 1.09, 95% CI 0.85–1.40; p = 0.189 for heterogeneity; I^2^ = 39.9%) or USA (RR 1.28, 95% CI 0.90–1.81; p<0.001 for heterogeneity; I^2^ = 88.6%). In further stratified analysis by the methods of ascertainment of diabetes, the summary RRs with 95% CIs were 1.34 (95% CI 1.11–1.62) for studies using self-report, and 1.11 (95% CI 0.95–1.31) for others methods.

**Table 3:** Subgroup analysis of relative risks for the association between diabetes and bladder cancer.

Subgroup	References		Heterogeneity test		
		RR (95% CI)	Q	P	I^2^ (%)
**The measure of relative risk**					
Standardized incidence ratio	16, 19, 33–35	1.01 (0.82, 1.24)	47.44	<0.001	87.4
Rate ratio or hazard ratio	12–15, 17, 18, 20, 21, 31, 32	1.19 (1.04, 1.36)	57.74	<0.001	82.7
**Geographical region**					
Europe	14, 15, 21	1.09 (0.85, 1.40)	3.33	0.189	39.9
USA	17, 18, 32	1.28 (0.90, 1.81)	17.47	<0.001	88.6
Asia	12, 13, 20, 31	1.21 (1.15, 1.28)	2.43	0.658	0.0
**Adjustment for more than three confounders**					
Yes	12–14, 17, 18, 20, 32	1.20 (1.02, 1.42)	48.45	<0.001	85.6
No	15, 21, 31	1.17 (0.94, 1.47)	4.62	0.099	56.7
**Adjustment for smoking**					
Yes	12–14, 18, 31, 32	1.32 (1.18, 1.49)	5.62	0.467	0.0
No	15, 17, 20, 21	1.07 (0.90, 1.27)	38.22	<0.001	92.2
**Diabetes ascertainment**					
Self-report	12–14, 18, 32	1.34 (1.11, 1.62)	5.62	0.345	11.0
Others methods	15, 17, 20, 21, 31	1.11 (0.95, 1.31)	44.32	<0.001	91.0

RR, relative risk; CI, confidence interval.

We also investigated the impact of confounding factors on the estimates of relative risk (Table 3). Cigarette smoking is a risk factor for both diabetes and bladder cancer, and thus a potential confounder of the relationship between diabetes and risk of bladder cancer. Among the six studies that controlled for cigarette smoking, the pooled RR was 1.32 (95% CI 1.18–1.49; p = 0.467 for heterogeneity; I^2^ = 0%). Moreover, some studies in our analysis adjusted for more than three confounders. Therefore, we examined if more thoroughly adjusting for potential confounders affected the pooled RR and degree of heterogeneity (Table 3). The effect estimate for studies that adjusted for more than three confounders was RR, 1.20 (95% CI 1.02–1.42; p<0.001 for heterogeneity; I^2^ = 85.6%).

### Publication Bias

**Figure 3:**
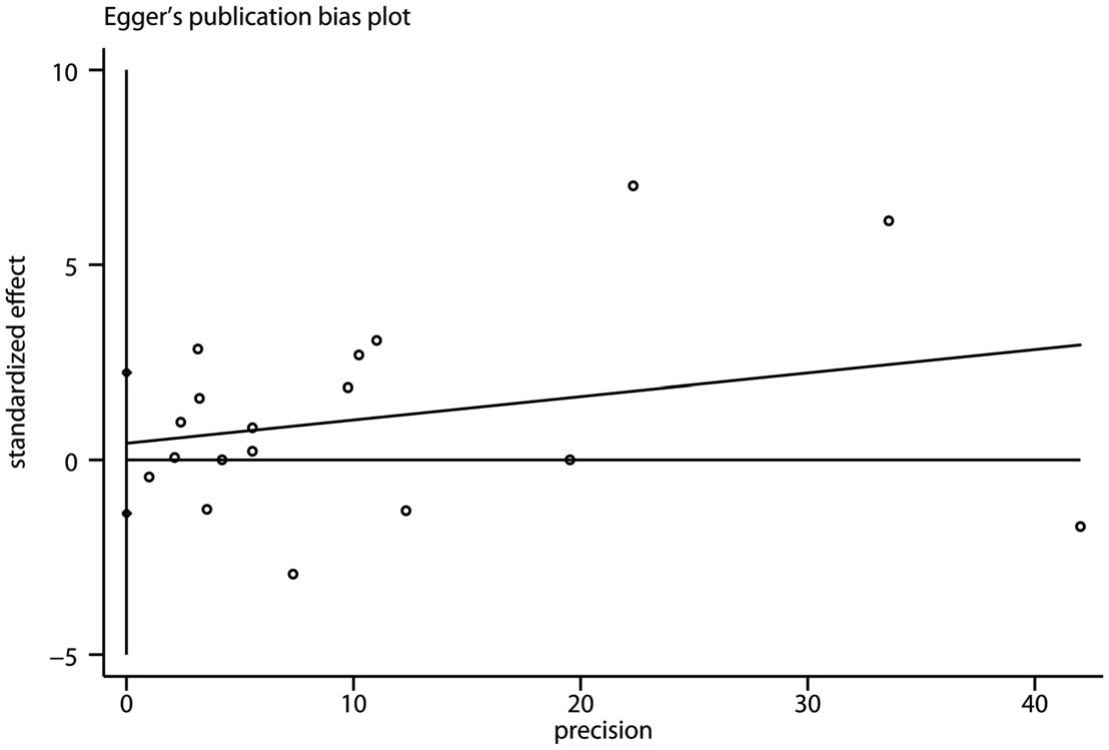
Funnel plot of cohort studies evaluating the association between diabetes and bladder cancer. Egger’s regression asymmetry test (p = 0.62). Standardized effect was defined as the odds ratio divided by its standard error. Precision was defined as the inverse of the standard error.

There was no evidence of significant publication bias either with the Begg’s test (P = 0.76) or with Egger’s test (Fig. 3, P = 0.62).

## Discussion

The findings of this meta-analysis of fifteen cohort studies indicate that diabetes is associated with an 11% increased risk of bladder cancer. It tended to be more remarkable for studies with a rate ratio or hazard ratio as the measure of relative risk than for studies with a standardized incidence ratio.

At present, whether diabetes is independently associated with incidence of bladder cancer remains controversial. Results from our subgroup analysis restricted to studies with control for smoking or adjusted for more than three confounders were more robust than that reported in the overall analysis, which indicated that the association may have been diluted by poor study methodologies and diabetes is probably an independent risk factor of bladder cancer.

Of note, the association between diabetes and bladder cancer was more pronounced in studies with a rate ratio or hazard ratio as the measure of relative risk than in studies with a standardized incidence ratio. Studies using standardized incidence ratio and standardized mortality ratio to estimate the relative risk may underestimate the true relative risk [36], [37]. Because if the general population is taken to represent unexposed persons, it is almost inevitably biased in that it comprises all types of people including exposed ones [37]. Therefore, the summary RRs risk of this meta-analysis may have been attenuated by the results from studies using standardized incidence ratio as the measure of relative risk and the results of our meta-analysis were actually statistically robust.

The association between the duration of diabetes and risk of bladder cancer have been assessed in some studies, and inconsistent results were found [12], [13], [15]–[17], [20], [34]. In the study by Hemminki et al. [16], the standardized incidence ratio of bladder cancer declined from 1.37 with no latency period to 0.96 for a 5 year latency period, while Ogunleye et al. [15] reported that the RR between diabetes and bladder cancer was 0.70 (95% CI 0.40–1.21) when including all cases and 0.53 (95% CI 0.24–1.15) when excluding cases diagnosed within the first year of follow-up. The study conducted by Atchison et al. [17] also suggested the risk of bladder cancer declined over time. However, another three reports [12], [13], [20] found higher relative risks after excluding the first 2, 3.5 or 5 years of follow-up. Because of the inconsistent results, it remains unclear whether the duration of diabetes is directly associated with the risk of bladder cancer. Interestingly, we noticed that the studies reported risk reduction over time were conducted in western countries, while higher relative risks for longer duration were suggested by studies from Asian countries. The mechanism behind this difference is not clear and should be further studied in the future.

A relationship between diabetes and risk of bladder cancer is biologically plausible. Type 2 diabetes is associated with insulin resistance, compensatory hyper-insulinemia, and up-regulated level of IGF-1. IGF-1 could stimulate cell proliferation and inhibit apoptosis. Several epidemiological studies have implicated IGF-I in the development of breast and colorectal cancers [38], [39]. A USA case-control study also has found statistically significantly higher circulating levels of IGF-I in bladder cancer cases than in controls [40]. An important role of IGF-I in the development of bladder cancer is also supported by studies in animals [41]. Additionally, diabetes is also associated with an increased risk of urinary tract infection [42] and urinary tract calculi [43], which have been related to various histologic types of bladder cancer, including transitional cell carcinoma, the predominant type [44], [45].

Substantial heterogeneity was observed among studies of diabetes and bladder cancer risk, which may be due to different adjustment for confounding factors and different mixtures of type 1 and type 2 diabetic patients. Some studies included in this meta-analysis did not distinguish between type 1 and type 2 diabetes. As type-1 diabetes may not be related to bladder cancer risk [29], [30], different proportions of type 1 and type 2 diabetic participants in the studies may in part account for the observed heterogeneity.

A major strength of our study is that with the accumulating evidence and enlarged sample size, we have enhanced statistical power to derive a more precise and reliable estimation of the relationship between diabetes and bladder cancer risk. Nonetheless, some limitations should be mentioned. One potential limitation of this meta-analysis was the various assessments of diabetes used between studies. Some studies used self-report as the method of diabetes ascertainment, which may lead to some misclassification of diabetic persons as non-diabetic persons. This underreporting may result in an underestimate of the magnitude of the association between diabetes and bladder cancer risk. However, earlier studies have suggested that self-reported diabetes have good agreement with medical records [46], [47]. A second limitation is the uncontrolled or unmeasured risk factors potentially produce biases. Although the magnitude of increased risk reported in studies adjusted for more than three confounders was more robust than that reported in the overall analysis, we still cannot rule out the possibility that residual confounding could affect the results. Recently, some studies reported that thiazolidinediones, particularly pioglitazone, were associated with an increased risk of bladder cancer [48], [49]. However, most of the studies included in this meta-analysis did not provide the information of oral hypoglycemic use. Thus we failed to evaluate the therapeutic agents’ influence on the association between the diabetes and bladder cancer risk. Finally, in any meta-analysis, the possibility of publication bias is of concern, because small studies with null results tend not to be published. However, we found no evidence of publication bias in this meta-analysis.

In summary, our findings support that diabetes was associated with the increased risk of bladder cancer. More future studies are warranted to get a better understanding of the association and to provide convincing evidence for clinical practice in bladder cancer prevention.
